# Supplementary figures and images for: Anchialine pool shrimp (Halocaridina rubra) as an indicator of sewage in coastal groundwater ecosystems on the island of Hawaiʻi
Source: PLoS One. 2023 Aug 31;18(8):e0290658. doi: 10.1371/journal.pone.0290658 (PMC10470924; doi:10.1371/journal.pone.0290658)

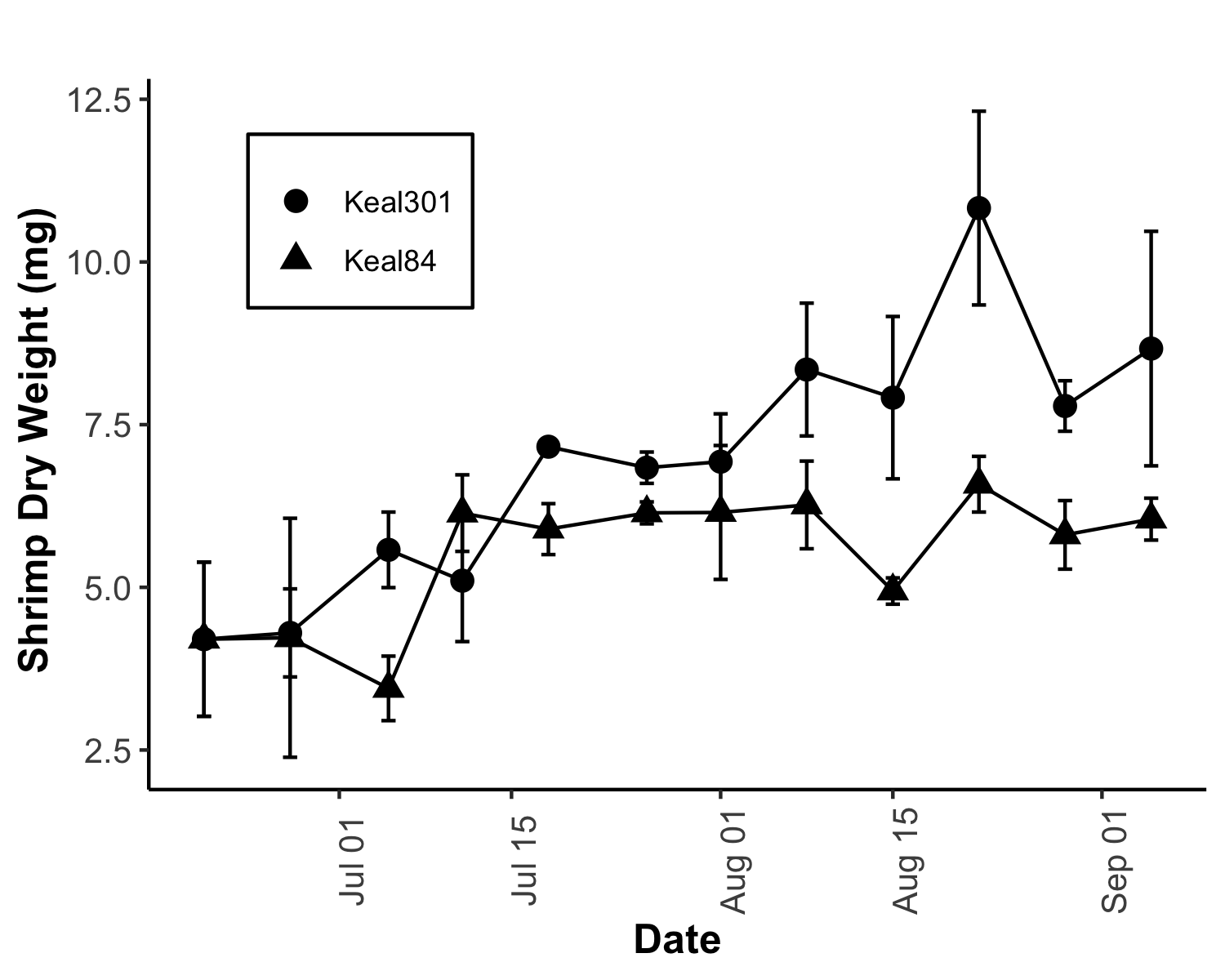

Supplement: S1 Fig — Error bars are the standard deviation. Pool Keal84 is the control treatment and pool Keal301 is the sewage influenced pool. (TIF) [file pone.0290658.s001.tif]
