# Supplementary material for: Anchialine pool shrimp (Halocaridina rubra) as an indicator of sewage in coastal groundwater ecosystems on the island of Hawaiʻi
Source: PLoS One. 2023 Aug 31;18(8):e0290658. doi: 10.1371/journal.pone.0290658 (PMC10470924; doi:10.1371/journal.pone.0290658)
Supplement: S1 Table — Location is the island-wide naming convention for pools (‘Island_land division_number’). ID is a shortened version used in this paper. Year of sampling is indicated by an x. (DOCX) [file pone.0290658.s002.docx]

**Table S1: Anchialine pools where *H. rubra* were collected for δ ^15^N analysis between 2015 to 2017.** Location is the island-wide naming convention for pools (‘Island_land division_number’). ID is a shortened version used in this paper. Year of sampling is indicated by an x.

| **Location** | **ID** | **2015** | **2016** | **2017** |
| --- | --- | --- | --- | --- |
| HA_Lalami_001 | Lala01 | x |  |  |
| HA_Puuana_015 | Puua15 | x |  |  |
| HA_Puuana_065 | Puua65 | x |  |  |
| HA_Puuana_071 | Puua71 | x |  |  |
| HA_Puuwaa_015 | Puuw15 | x |  |  |
| HA_Puuwaa_019 | Puuw19 | x |  |  |
| HA_Kalaoa_007 | Kala07 | x |  |  |
| HA_Keaho_015 | Keah01 | x |  |  |
| HA_Kaloko_005 | Kalo05 | x | x | x |
| HA_Kaloko_053H | Kalo53 |  | x | x |
| HA_Kaloko_016 | Kalo16 |  | x | x |
| HA_Kaloko_120 | Kalo120 |  | x | x |
| HA_Kohana_144 | Koha144 |  | x | x |
| HA_Kaloko_146 | Kalo146 |  | x |  |
| HA_Kealak_096 | Keal96 |  | x | x |
| HA_Kealak_099 | Keal99 |  | x | x |
| HA_Kealak_084 | Keal84 |  | x | x |
| HA_Kealak_308 | Keal308 |  | x | x |
| HA_Kealak_305 | Keal305 | x | x | x |
| HA_Kealak_301 | Keal301 |  | x | x |
