# Supplementary material for: Anchialine pool shrimp (Halocaridina rubra) as an indicator of sewage in coastal groundwater ecosystems on the island of Hawaiʻi
Source: PLoS One. 2023 Aug 31;18(8):e0290658. doi: 10.1371/journal.pone.0290658 (PMC10470924; doi:10.1371/journal.pone.0290658)
Supplement: S2 Table — Stable isotopes were from shrimp tissues with the pools sampled. Standard deviation is shown with the mean (n = 3) for stable isotopes. (DOCX) [file pone.0290658.s003.docx]

**Table S2: Water quality characteristics and nutrients for 11 anchialine pools in and south of Kaloko-Honokōhau National Historical Park during 2016 and 2017.** Stable isotopes were from shrimp tissues with the pools sampled. Standard deviation is shown with the mean (n=3) for stable isotopes.

**2016 Results**

| **Location** | **TDN (mg/L)** | **NO_3_+NO_2_ (mg/L)** | **TDP (mg/L)** | **δ^15^N (‰)** | **δ^13^C (‰)** | **Salinity (PSS)** |
| --- | --- | --- | --- | --- | --- | --- |
| Kalo05 | 1.05 | 1.01 | 0.11 | 4.98 ± 0.6 | 19.97 ± 0.3 | 13.71 |
| Kalo53 | 1.50 | 1.44 | 0.13 | 4.65 ± 0.6 | 23.24 ± 0.4 | 9.17 |
| Kalo16 | 1.40 | 1.31 | 0.15 | 3.91 ± 0.5 | 20.48 ± 0.4 | 8.86 |
| Kalo120 | 0.65 | 0.57 | 0.08 | - | - | 15.63 |
| Koha144 | 1.10 | 0.91 | 0.08 | 7.42 ± 0.4 | 20.41 ± 0.6 | 11.71 |
| Kalo146 | 0.90 | 0.78 | 0.11 | 6.53 ± 0.3 | 20.87 ± 1.14 | 14.48 |
| Keal96 | 1.37 | 1.12 | 0.19 | 10.89 ± 1.4 | 15.00 ± 1.92 | 15.43 |
| Keal99 | 1.25 | 1.09 | 0.16 | 8.73 ± 0.4 | 19.97 ± 0.9 | 14.82 |
| Keal84 | 0.29 | 0.13 | 0.05 | 8.97 ± 0.5 | 14.37 ± 1 | 23.66 |
| Keal308 | 5.62 | 5.49 | 1.22 | 21.49 ± 0.7 | 17.06 ± 0.5 | 15.19 |
| Keal305 | 6.04 | 5.62 | 1.22 | 22.59 ± 0.6 | 19.76 ± 0.7 | 15.1 |
| Keal301 | 5.02 | 4.89 | 1.15 | 24.68 ± 0.7 | 13.76 ± 1.2 | 15.61 |

**2017 Results**

| **Location** | **TDN (mg/L)** | **NO_3_+NO_2_ (mg/L)** | **TDP (mg/L)** | **δ^15^N (‰)** | **δ^13^C (‰)** | **Salinity (PSS)** |
| --- | --- | --- | --- | --- | --- | --- |
| Kalo05 | 1.00 | 0.96 | 0.13 | 5.05 ± 0.4 | 19.08 ± 0.9 | 13.5 |
| Kalo53 | 1.33 | 1.27 | 0.13 | 4.74 ± 0.5 | 23.21 ± 1.5 | 9.04 |
| Kalo16 | 1.39 | 1.10 | 0.12 | 4.20 ± 0.8 | 21.95 ± 0.7 | 8.92 |
| Kalo120 | 0.86 | 0.75 | 0.08 | 8.34 ± 1.1 | 21.56 ± 0.6 | 15.12 |
| Koha144 | 1.68 | 1.58 | 0.12 | 8.09 ± 0.5 | 20.17 ± 1.6 | 11.35 |
| Kalo146 | - | - | - | - | - | - |
| Keal96 | 0.87 | 0.74 | 0.15 | 10.51 ± 0.2 | 16.60 ± 1.6 | 14.9 |
| Keal99 | 0.79 | 0.62 | 0.15 | 8.72 ± 1 | 21.05 ± 1.0 | 14.45 |
| Keal84 | 0.15 | 0.02 | 0.02 | 9.20 ± 0.3 | 14.18 ± 0.8 | 22.56 |
| Keal308 | 2.71 | 1.85 | 1.24 | 21.55 ± 0.8 | 18.59 ± 0.7 | 15.17 |
| Keal305 | 2.90 | 1.84 | 1.26 | 22.98 ± 0.4 | 19.40 ± 0.8 | 15.22 |
| Keal301 | 2.69 | 1.80 | 1.17 | 23.58 ± 1.5 | 15.82 ± 1.9 | 15.65 |
