# Supplementary material for: Anchialine pool shrimp (Halocaridina rubra) as an indicator of sewage in coastal groundwater ecosystems on the island of Hawaiʻi
Source: PLoS One. 2023 Aug 31;18(8):e0290658. doi: 10.1371/journal.pone.0290658 (PMC10470924; doi:10.1371/journal.pone.0290658)
Supplement: S3 Table — Water for the sewage exposed pool treatments (301) was collected at that pool. Pool for the control treatment was collected at pool 84. (DOCX) [file pone.0290658.s004.docx]

**Table S3: Mean values of water quality measures in mesocosm containers (mean ± standard deviation; n = 3) at the start of the experiment, at day 50, at day 50 after the new water was placed in the mesocosm, and at day 77 at the end of the experiment.** Water for the sewage exposed pool treatments (301) was collected at that pool. Pool for the control treatment was collected at pool 84.

| **Nutrient (mg/L)** | **Treatment** | **Start** | **Day 50** | **Day 50 - New** | **Day 77** |
| --- | --- | --- | --- | --- | --- |
| **NO_3_ + NO_2_** | **control** | 0.22 ± 0 | <0.001 | 0.001 ± 001 | 0.002 ± 0.002 |
|  | **sewage** | 1.35 ± 0.02 | 0.01 ± 0.01 | 1.22 ± 0.08 | 0.02 ± 0.01 |
| **NH_4_** | **control** | 0.05 ± 0.01 | 0.02 ± 0.01 | <0.001 | 0.12 ± 0.19 |
|  | **sewage** | 0.06 ± 0 | 0.02 ± 0.0 | 0.03 ± 0 | 0.01 ± 0 |
| **TDP** | **control** | 0.06 ± 0 | 0.07 ± 0.01 | 0.02 ± 0 | 0.08 ± 0.03 |
|  | **sewage** | 1.11 ± 0.01 | 1.13 ± 0.1 | 1.13 ± 0.05 | 1.16 ± 0.17 |
| **TDN** | **control** | 0.39 ± 0.02 | 1.21 ± 0.05 | 0.2 ± 0.01 | 0.58 ± 0.02 |
|  | **sewage** | 1.47 ± 0.01 | 1.42 ± 0.04 | 1.27 ± 0.08 | 0.63 ± 0.08 |
