# Supplementary material for: Anchialine pool shrimp (Halocaridina rubra) as an indicator of sewage in coastal groundwater ecosystems on the island of Hawaiʻi
Source: PLoS One. 2023 Aug 31;18(8):e0290658. doi: 10.1371/journal.pone.0290658 (PMC10470924; doi:10.1371/journal.pone.0290658)
Supplement: S1 Appendix — Files are available on the NPS- IRMA website (https://doi.org/10.57830/2299671). (DOCX) [file pone.0290658.s005.docx]

**Appendix S1: Raw Data Tables**

**Raw data tables are located at the following Data repository. Examples of the tables are below.**

Marrack L and Beavers SC. 2023. Dataset for the project: Anchialine pool shrimp (Halocaridina rubra) as an indicator of sewage in coastal groundwater ecosystems on the island of Hawaiʻi. National Park Service. <https://doi.org/10.57830/2299671>

1. **SUPP_mesocosm2018.xlsx:** Mesocosm experiment data for shrimp. Fields include pool fed from, sample date, day of experiment (Day), whole dried weight of shrimp, and nitrogen and carbon isotope values.

**Example of format:**

| **Sample#** | **SampleID** | **Pool** | **Date** | **Day** | **Whole_wt**  **(mg)** | **δ^15^N (‰)** | **δ^13^C (‰)** |
| --- | --- | --- | --- | --- | --- | --- | --- |
| 5 | 84B.6.20 | HA_Kealak_301 | 6/20/18 | 1 | 5.123 | 9.17 | -14.68 |
| 41 | 84C.6.20 | HA_Kealak_301 | 6/20/18 | 1 | 5.634 | 8.82 | -14.49 |
| 63 | 84A.6.20 | HA_Kealak_301 | 6/20/18 | 1 | 1.852 | 8.4 | -14.86 |
| 6 | 301C.6.27 | HA_Kealak_301 | 6/27/18 | 7 | 2.986 | 11.06 | -16.04 |
| 32 | 301A.6.27 | HA_Kealak_301 | 6/27/18 | 7 | 4.675 | 12.1 | -13.5 |
| 44 | 301B.6.27 | HA_Kealak_301 | 6/27/18 | 7 | 5.238 | 11.74 | -12.72 |
| 65 | 301B.7.5 | HA_Kealak_301 | 7/5/18 | 15 | 4.491 | 12.15 | -18.59 |
| 76 | 301C.7.5 | HA_Kealak_301 | 7/5/18 | 15 | 6.475 | 12.95 | -14.54 |
| 88 | 301A.7.05 | HA_Kealak_301 | 7/5/18 | 15 | 5.763 | 13.7 | -14.49 |

1. **SUPP_WQ_Analysis_KAHO&Meso.xlsx :** Water quality results from lab. Includes data for 2016 and 2017 at Kaloko-Honokōhau National Historical Park pool locations as well as throughout the mesocosm experiment.

**Example of format:**

**
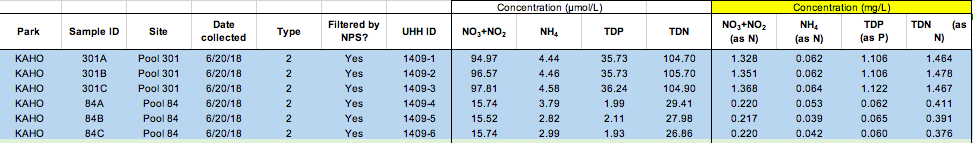
**

1. **SUPP_KAHO_2016_2017_Isotope.xlsx :** Stable isotope data for individual shrimp collected in 2016 and 2017 at Kaloko-Honokōhau National Historical Park pool locations.

**Example of format:**

**
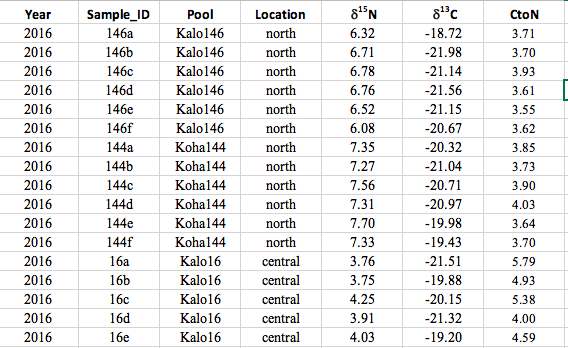
**

1. **SUPP_WHawaii_2015_isotope.xlsx :** Stable isotope data for individual shrimp collected in 2015 in West Hawaii.

**Example of format:**

**
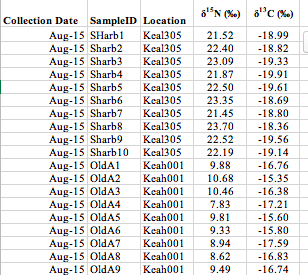
**
