# Supplementary material for: Anchialine pool shrimp (Halocaridina rubra) as an indicator of sewage in coastal groundwater ecosystems on the island of Hawaiʻi
Source: PLoS One. 2023 Aug 31;18(8):e0290658. doi: 10.1371/journal.pone.0290658 (PMC10470924; doi:10.1371/journal.pone.0290658)
Supplement: Attachment — Submitted filename: ResponsetoReviewers_PLOSONE.docx [file pone.0290658.s006.docx]

"Lisa C Marrack" lisamarrack@gmail.com
PLOS ONE Decision: Revision required [PONE-D-23-04906]

**PONE-D-23-04906
Title:** Anchialine pool shrimp (*Halocaridina rubra*) as an indicator of sewage in coastal groundwater ecosystems on the island of Hawaiʻi

Dear Editors:

We appreciate the positive response to our manuscript and the helpful comments from reviewers. We have responded to all of the requests to update the manuscript and each request/change is detailed below. These changes are visible in the “Revised Manuscript with Track Changes” file.

A revised Supplemental document was uploaded – it has corrected S1 Fig and Table labels.

We have created a data repository in IRMA (the National Park Data center). The citation is in the supplemental document. Is there another place it should be?

Marrack L and Beavers SC. 2023. Dataset for the project: Anchialine pool shrimp (Halocaridina rubra) as an indicator of sewage in coastal groundwater ecosystems on the island of Hawaiʻi.. National Park Service. [https://doi.org/10.57830/2299671](https://doi.org/10.57830/2299671)

Please let us know if there are any further questions.

Best regards,

Lisa Marrack & Sallie Beavers

_____________________________________________________________________________

1. *Please ensure that your manuscript meets PLOS ONE's style requirements, including those for file naming. The PLOS ONE style templates can be found at*

*https://journals.plos.org/plosone/s/file?id=wjVg/PLOSOne_formatting_sample_main_body.pdf*

*https://journals.plos.org/plosone/s/file?id=ba62/PLOSOne_formatting_sample_title_authors_affiliations.pdf*

Changed format for main body paragraph titles and figure/table captions.

Changed format for author affiliations.

*2. We note that the grant information you provided in the ‘Funding Information’ and ‘Financial Disclosure’ sections do not match. When you resubmit, please ensure that you provide the correct grant numbers for the awards you received for your study in the ‘Funding Information’ section.*

The **Financial Disclosure statement** correctly states that “The authors received no specific funding for this work “.

The only place we could find potentially conflicting information for “Funding information” was in the **Author Contributio**n section which said “Both LM and SB were responsible for funding acquisition”.

We delete the statement “Both LM and SB were responsible for funding acquisition”. We had no specific grant that covered this work.

*3. We note that Figure 1 in your submission contain [map/satellite] images which may be copyrighted.*

Figure 1 was updated so that the lat/long coordinates are now visible and the scale bars are both in the same units (KM). Additionally, the layers that are not our own are now cited with the correct citation. For (a) the entire island inset – the DEM model is in the public domain via the USGS (see reference). For the Parcels-TMK - Hawaii county (zone 7); Roads - Hawaii county (zone 7 highways); and Onsite Sewage Disposal Systems, a letter of permission was obtained and attached. These are also available through a public portal (see references).

**Fig 1 References**

U.S. Geological Survey. USGS 10-m Digital Elevation Model (DEM): Hawaii: Big Island. [Accessed 2021] Available at: http://pacioos.org/metadata/usgs_dem_10m_bigisland.html; 2015.

County of Hawaii Planning Department. TMK Plats for the State of Hawaii as of May, 2018. [Accessed 2021] Available at: https://geoportal.hawaii.gov/datasets/HiStateGIS::parcels-tmk-plat/explore; 2018.

County of Hawaii Planning Department. Hawaii County Major Roads as of 2005. [Accessed 2021] Available at: https://geoportal.hawaii.gov/datasets/HiStateGIS::major-roads-hawaii-county/explore?location=19.735164%2C-155.787845%2C11.00; 2014.

State of Hawaii Department of Health. On-site Sewage Disposal Systems for the island of Hawaii, as of 2010. [Accessed 2021] Available at: https://geoportal.hawaii.gov/datasets/HiStateGIS; 2017.

*4. Please review your reference list to ensure that it is complete and correct. Any changes to the reference list should be mentioned in the rebuttal letter that accompanies your revised manuscript. If you need to cite a retracted article, indicate the article’s retracted status in the References list and also include a citation and full reference for the retraction notice.*

References were reviewed for formatting and corrected where needed. Four references were added for the Figure 1 data layers (see above) and 3 were added to the introduction as was requested by Reviewer 2 ( see list below). The Reference list was renumbered appropriately.

*5. Specific comments from reviewers were addressed/corrected in the manuscript*

a. In Abstract: Replace “ranged between 2.74 and 58 22.46‰.” with “ranged between 2.74‰ and 58 22.46‰.”
b. Include value “The significant positive relationship between δ15N and dissolved nitrogen and phosphorus”
c. Include methodology (sample testing technique and data analysis) and a key recommendation in Abstract.
d. Include/replace references of recent studies of last three years in introduction and discussions part.
e. The start of “Conclusions part” with suggestions is not a good idea. Give conclusions of your study avoiding references
f. Add coordinates in figure-1. Use uniform unit for scale bar in figure-1
g. Reviewer #2: The manuscript is very impressive and covers all data including statistical evaluations in detail. However a minor addition of a paragraph highlighting how quality assurance was undertaken will be appreciated (**see paragraph below**). One or two very recent (2021/2022) relevant citations in the introduction part will make the manuscript amazing.

**QA/QC**

The quality assessment and control measures used in the water sampling portion of this study were developed by the National Park Service Pacific Island Network’s water quality monitoring program [32]. These measures include calibrating the YSI sonde prior to field measurements, collecting water samples at an outgoing tide, collecting water samples in triplicate within previously acid-washed bottles at each pool, and freezing water samples prior to nutrient analysis. For the shrimp tissue samples, replicate samples were collected at each pool or mesocosm group within minutes of each other and were prepared as described in stable isotope analysis above. Water nutrient level analyses and shrimp tissue isotope analyses were conducted at independent laboratories with their internal QA/QC protocols.

**Recent citations added to Intro:**

Aguiar DK, Wiegner TN, Colbert SL, Burns J, Abaya L, Beets J, et al. Detection and impact of sewage pollution on South Kohala’s coral reefs, Hawai ‘i. Mar Pollut Bull. 2023;188: 114662.

Khan S, Naushad M, Govarthanan M, Iqbal J, Alfadul SM. Emerging contaminants of high concern for the environment: Current trends and future research. Environ Res. 2022;207: 112609.

Santos IR, Chen X, Lecher AL, Sawyer AH, Moosdorf N, Rodellas V, et al. Submarine groundwater discharge impacts on coastal nutrient biogeochemistry. Nat Rev Earth Environ. 2021;2: 307–323.
